# Swim with the tide: Tactics to maximize prey detection by a specialist predator, the greater sea snake (*Hydrophis major*)

**DOI:** 10.1371/journal.pone.0239920

**Published:** 2020-10-01

**Authors:** Vinay Udyawer, Claire Goiran, Olivier Chateau, Richard Shine

**Affiliations:** 1 Australian Institute of Marine Science, Darwin, Northern Territory, Australia; 2 LabEx Corail & ISEA, Université de la Nouvelle-Calédonie, Nouméa Cedex, New Caledonia; 3 Laboratory of Marine Biology and Ecology, Aquarium des Lagons, Nouméa, New Caledonia; 4 Department of Biological Sciences, Macquarie University, Sydney, New South Wales, Australia; University of Regina, CANADA

## Abstract

The fitness of a predator depends upon its ability to locate and capture prey; and thus, increasing dietary specialization should favor the evolution of species-specific foraging tactics tuned to taxon-specific habitats and cues. Within marine environments, prey detectability (e.g., via visual or chemical cues) is affected by environmental conditions (e.g., water clarity and tidal flow), such that specialist predators would be expected to synchronize their foraging activity with cyclic variation in such conditions. In the present study, we combined behavioral-ecology experiments on captive sea snakes and their prey (catfish) with acoustic tracking of free-ranging sea snakes, to explore the use of waterborne chemical cues in this predator-prey interaction. In coral-reef ecosystems of New Caledonia, the greater sea snake (*Hydrophis major*) feeds only upon striped eel catfish (*Plotosus lineatus*). Captive snakes became more active after exposure to waterborne chemical cues from catfish, whereas catfish did not avoid chemical cues from snakes. Movement patterns of tracked snakes showed that individuals were most active on a rapidly falling tide, which is the time when chemical cues from hidden catfish are likely to be most readily available to a foraging predator. By synchronizing foraging effort with the tidal cycle, greater sea snakes may be able to exploit the availability of chemical cues during a rapidly falling tide to maximize efficiency in locating and capturing prey.

## 1. Introduction

Predators and their prey interact via a range of sensory modalities [1]. For example, some predators (e.g., eagles) and prey (e.g., antelope) are renowned for their visual acuity [2]. Other taxa utilize auditory cues to locate potential meals or threats [3, 4]. Yet others rely upon vibrations transmitted through webs [5], water [6, 7], the ground [8], or faint electrical signals [9]. One of the most widespread modes of detection comprises chemical cues [10]. Many predators locate prey items by chemical cues, and many prey taxa avoid the chemicals of predators [11, 12].

Features of the local environment strongly influence the effectiveness of specific channels of communication. Thus, for example, hunting felids typically approach large prey items upwind, thereby eliminating chemical cues that would reveal their presence to the prey [13]. As a result, such predators may hunt most often at times and in places when winds are stable in direction rather than eddying. Likewise, some types of substrates retain chemical cues longer than others, and/or facilitate long-distance following of substrate-deposited trails [14]. Background noise levels can reduce the ability of a predator to locate its prey, or a prey item to detect a predator’s approach [15].

To understand predator-prey interactions, we need to identify what types of cues each participant uses to locate the other and to explore how local environments influence the availability of such cues. Although studies on these questions have been conducted in many systems (see above), little attention has been paid to others. For example, an extensive literature explores the use of substrate-deposited chemical cues by terrestrial snakes [16], but marine snakes have attracted less research. Aquatic habitats largely eliminate substrate-deposited chemical trails, promoting reliance on other types of cues (such as visual ones [17]). However, many marine organisms (including fish) produce waterborne chemicals that may be detectable by a snake’s highly specialized vomeronasal system [18, 19], and three species of sea snakes have been reported to use chemical cues from their prey to select foraging sites (*Emydocephalus annulatus* [20]; *Hydrophis melanocephalus* and *H*. *ornatus* [21]. Thus, although the three-dimensional nature of the aquatic environment reduces the usefulness of substrate-deposited cues, it allows other kinds of chemical cues to be widely disseminated.

In the present study, we describe a preliminary investigation of aspects of the predator-prey interaction between a marine snake and its primary prey, a catfish. Because these fish travel in swarms, and maintain cohesion within a swarm by producing chemical cues [22], we predicted that snakes would be able to detect such cues, and would increase foraging effort in response. The snakes are a major threat for these catfish (whose toxicity and sharp defensive spines deter most other predators), so we predicted that the fish might also benefit from detecting the approach of a snake. Lastly, if snakes rely upon waterborne chemical cues, we predicted that snakes will forage most intensely during the phase of the tidal cycle when such cues are most available: that is, on a rapidly falling tide. During the falling tide, water flows out from coral and rock crevices (where catfish shelter) into the broader water column, increasing the accessibility of chemical cues for a foraging snake. The availability of such cues likely would be lower when the tide ceases to flow, or when the incoming tide brings in a large volume of water that dilutes the chemicals produced by swarms of catfish. In this study, we test these ideas using straightforward experimental studies on responses of sea snakes and catfish to chemical cues from the other species, combined with information from acoustic telemetry on movement patterns of free-ranging snakes as a function of tidal phase.

## 2. Materials and methods

### 2.1. Study species and area

**Fig 1:**
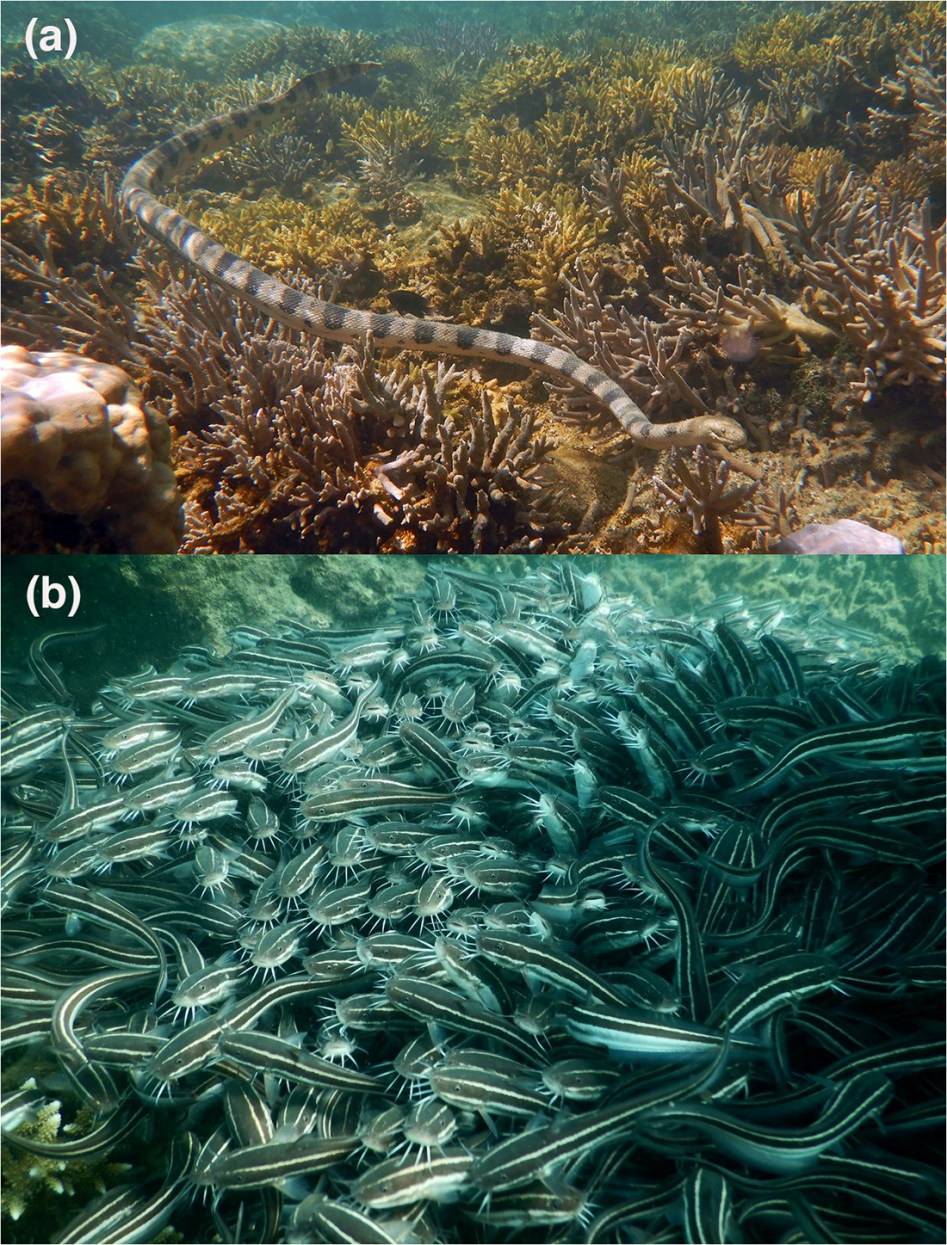
Study species. (a) Greater sea snake, *Hydrophis major*; (b) swarm of striped eel catfish, *Plotosus lineatus*. Photographs by (a) Claire Goiran, and (b) Aline Guémas.

The greater sea snake or olive-headed sea snake (*Hydrophis major*) is a fully aquatic hydrophiine elapid snake that is relatively heavy-bodied and can exceed 1.5 m in length [23] (Fig 1a). It is found in shallow to deep waters from the coasts of Western Australia to New Caledonia [24]. Published accounts are inconsistent with respect to dietary habits of these actively foraging mesopredators: older records suggest that a diverse array of fish species are consumed, whilst recent research from New Caledonia has reported only a single species in the diet (the striped eel catfish *Plotosus lineatus* [25, 26]; Fig 1b). The behavioral ecology of interactions between sea snakes and catfish remain unclear, but may involve predation both on solitary fish and on individuals in large swarms (see S1 Video). Although adults of this catfish species tend to be solitary, juvenile catfish form dense swarms that move about together while foraging, sometimes traveling hundreds of meters per hour, and hide together in crevices within the reef [27]. These aggregations are maintained by waterborne chemical cues [22].

The Baie des Citrons and Anse Vata (22°16’S, 166°26’E) are shallow bays beside the city of Noumea, in the Pacific archipelago of New Caledonia. The substrate is a mosaic of coral reef, sand, rocks and rubble [28]. While snorkeling at these sites, we often encounter *H*. *major* swimming rapidly in 1–2 m of water, tongue-flicking crevices in the coral and between boulders as they travel [29].

### 2.2. Methods for behavioral experiments

The present work is preliminary only. Sample sizes are small as a consequence of the difficulties of working with large and uncommon venomous snakes. The present study provides a basis from which to refine more detailed research questions.

**Fig 2:**
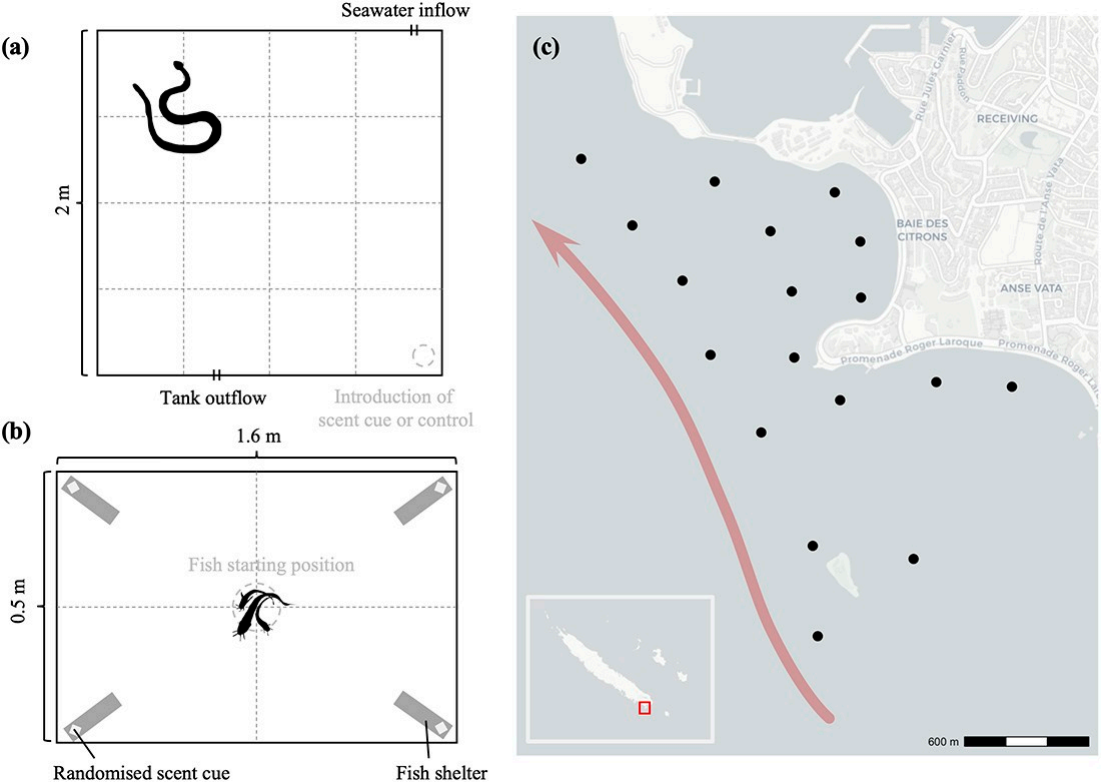
Behavioral trials and acoustic telemetry. (a) Laboratory setup for trials to quantify behavioral responses of four greater sea snakes, *Hydrophis major*, to waterborne chemical cues from striped eel catfish, *Plotosus lineatus*. (b) Laboratory setup for trials used to quantify behavioral responses of striped eel catfish, *Plotosus lineatus* to chemical cues of greater sea snakes, *Hydrophis major*. (c) Array of acoustic receivers (black points) used to track movements of 14 greater sea snakes over 349 days within the Baie des Citrons and Anse Vata, Noumea, New Caledonia. Arrow direction indicates predominant water-flow direction within the study site.

We captured four *H*. *major* (snout-vent length [SVL] 53–130 cm, mass 44–1150 g) from the Baie des Citrons and Anse Vata in January-March 2020, and maintained them for periods of 6 to 19 days in a large (2 × 2 m, 18 cm deep) tank with circulating seawater at the Aquarium des Lagons. During those periods, we conducted trials on the responses of snakes to chemical cues from catfish. To quantify distances moved by snakes, we defined 16 equally sized 0.5-m^2^ quadrats on the floor of the tank by stretching two sets of cords, perpendicular to each other, across the tank (Fig 2a). As a measure of activity, we scored the number of times a snake’s head crossed a cord within a 2-minute period after we introduced either chemical cues from fish, or a control stimulus. Where possible, we also scored tongue-flicking rates. The donors of those fish chemicals were a swarm of nine *Plotosus lineatus* (body lengths 7.5 to 12.5 cm), captured from nearby bays by staff at the Aquarium des Lagons and maintained at the Aquarium in a tank containing 30 L of seawater. At the beginning of a trial we moved a single snake from its holding tank into the large experimental tank, waited 15 min to allow the animal to acclimate and settle down, then took 10 L of water from the catfish tank and gently poured it into one corner of the large tank. For control trials, we took the same volume of water from an empty tank, and poured it into the test arena in the same way. Because the experimental tank had circulating seawater, chemical cues from each experiment were removed between successive trials by opening both the inlet and outlet valves of the tank to allow water replacement. Trials with different snakes were separated by 15 to 30 min, with each trial commencing after the focal snake had been inactive for at least 1 min. The order in which the stimuli were presented (control versus fish-scented water) was randomized. All trials were conducted during daylight hours (1000–1700 h).

To quantify responses of catfish to chemical cues from snakes, we used the same fish as described above, and conducted behavioral trials in the home tank within which fish were held. We added four plastic tubes (5 cm diameter, 15 cm long) as shelters, one to each corner of the catfish’s home tank (160 × 50 × 50 cm) with the open ends of the tubes all pointing towards the center of the tank (Fig 2b). Inside each shelter, we placed a square piece of cheesecloth. Two of those squares had been moistened and then vigorously rubbed along the body of a live *H*. *major* for 30 s, whereas two served as controls (moistened but not rubbed on a snake). For each trial, the two scented squares of cheesecloth were rubbed on different snakes each time [30]. After 5 min, we scored which shelter had been chosen as a retreat, lifted the tubes out of the tank, and replaced them in the same positions. The catfish inside the tube were gently poured back into the tank before the tube was replaced in position. After another 5 min, we scored which retreat had been chosen. After five such trials, we changed the locations of treatment (snake chemical) versus control shelters, then repeated the above procedure another five times at 5-minute intervals, then again changed the locations of shelters and repeated the trials five more times. In total, this experiment provided 15 replicates for catfish selection of shelter-sites as a function of the presence of chemical cues from sea snakes.

After the behavioral experiments were concluded, all snakes were fitted with acoustic transmitters as part of a larger study (see below), and released unharmed at their points of capture. Catfish were returned to the Aquarium for use in their public displays (the purpose for which they had originally been collected).

### 2.3. Telemetry methods

We hand-captured 19 *Hydrophis major* (15 males, 4 females; SVL range 63 to 125 cm) in the Baie des Citrons and Anse Vata in January and October 2017, and a qualified veterinarian surgically implanted acoustic transmitters (V9P-2H; Vemco Ltd., Bedford, Nova Scotia) into the snakes under gaseous anesthesia (for details see [31, 32]). Transmitters weighed <1% (mean ± SE; 0.65 ± 0.17%) of snake body mass and were neutrally buoyant; snake behavior and locomotor ability were assessed during a 24-hour post-surgery recovery period and appeared to be unaffected by the implantation. The animals were released at their sites of capture the day after surgery, and their movements over the following 349 days were monitored with an array of 18 acoustic receivers that recorded detections of individuals when they were within detection range of each receiver (for details about monitoring technique see [32]; Fig 2c). The detection range of receivers within the array was tested by comparing the expected and observed number of detections from range-testing tags placed at multiple locations within the array over a period of 1 week at the start of the study, and was measured to be on average 150 m. Receiver stations were arranged ~300 m apart through relatively shallow (<3 m depth) areas of the two bays, with all receivers remaining fully submerged and active across the full tidal range within the study site. We also obtained hourly data on tidal height (data from Service Hydrographique de la Marine Nationale [SHOM]) from which change in tidal height (henceforth hourly tidal range) and direction of flow (i.e., rising vs. falling tide) was calculated for each 60-minute observation period coinciding with movement records of telemetered snakes.

### 2.4. Statistical analysis

#### 2.4.1. Behavioral experiments

We used a linear mixed effects model using the ‘lme4’ software in R package [33] to compare snake activity levels (# of lines crossed in 2 min) between trials where we added catfish-scented water versus control water. Snake ID was included as a random effect, to control for the repeated measures nature of the analysis. For catfish responses to snake chemicals, we examined the number of times that the swarm of fish selected a snake-scented shelter versus a control shelter. We compared the proportion of choices the catfish swarm selected across all trials to that if no choice was made (0.5) using a Chi squared test in the R statistical environment [34]. We note that this retreat-site selection study was pseudoreplicated (same set of catfish used for each trial, same cheesecloth containing chemical cues in shelter-sites for successive trials) but we were constrained by availability of fish and equipment.

#### 2.4.2. Movements of free-ranging snakes

We first undertook a quality check of the raw detection data by identifying and removing erroneous detections based on the time and distance between consecutive detections within the array based on the procedure outlined by Hoenner et al. [35]. We calculated two descriptors of snake movements within each hour across the full monitoring period. The first was the number of movements detected for each tracked snake, and the second was the total distance moved by that snake. Movement metrics were then calculated by estimating total distances between consecutive detections within the hourly bins using the ‘VTrack’ package [36] in R [34]. Both variables were ln-transformed to improve normality of residuals. Hourly metrics of movement were then compared to hourly changes in tide heights. Changes in hourly tide heights were used as a proxy for tidal flow rates and to quantify flow direction (i.e., falling or rising tides), with larger differences between hourly tide heights indicative of periods of stronger tidal flow. We used linear mixed effects models to compare hourly tide height differences to the two metrics of snake movements, across seasons (i.e., summer [November–April] vs. winter [May–October]) and times of day (i.e., day vs. night) including snake ID as a random effect to control for pseudoreplication. Models were weighted by numbers of detections recorded during each hourly tidal height category for each individual to account for the unbalanced detection rates across the tidal range.

### 2.5. Ethics statement

Research was conducted using methods approved by the Charles Darwin University animal ethics committee (Permit A18029) and under collection permits from New Caledonia Province Sud 3252-2017/ARR/DENV.

## 3. Results

### 3.1. Behavioral experiments

**Fig 3:**
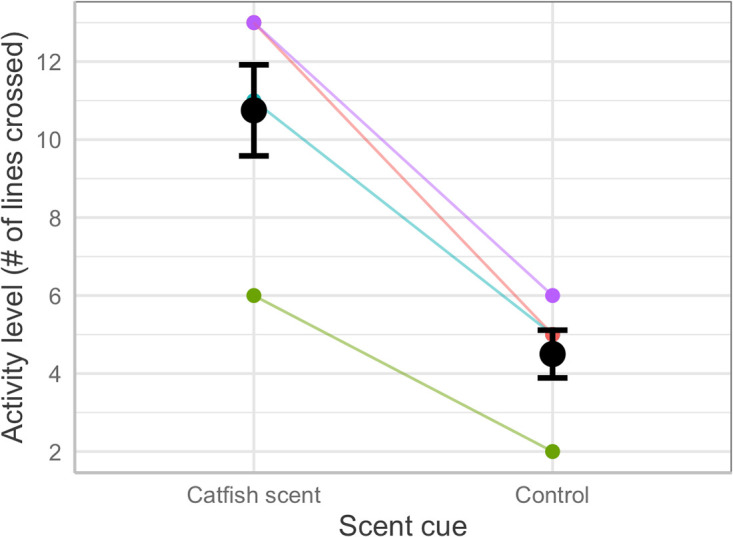
Snake activity. Activity levels (# of lines crossed) of captive greater sea snakes, *Hydrophis major*, in response to addition of water containing versus not containing chemical cues from striped eel catfish, *Plotosus lineatus*. Data for each snake are shown with colored lines, and overall means and standard errors are shown in black for each category.

In the 2-minute period following addition of cues to the tank, snakes were about twice as active if the water contained catfish chemical than if it did not (*F*_1,4_ = 53.57, *p* < 0.006; Fig 3). The increased activity was accompanied by high rates of extrusion of the tongue, as is typical of prey-searching snakes [37]. In trials of shelter-site selection, all nine catfish stayed together in all trials but the swarm did not choose the control-scented shelters more often than the snake-scented shelters (total choices 7 vs. 8 respectively; χ^2^ = 0.06, *p* = 0.79).

### 3.2. Movements of free-ranging snakes

**Fig 4:**
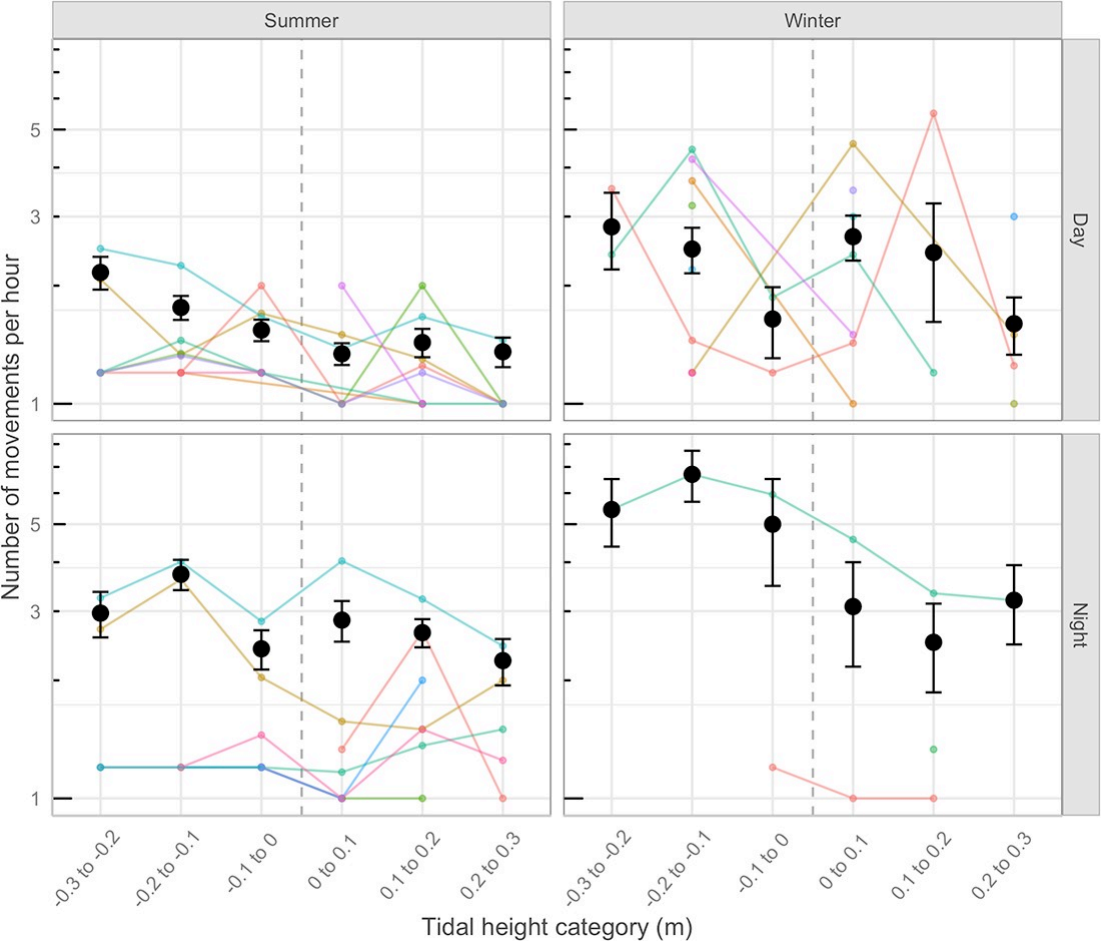
Snake movements. Number of hourly movements recorded by 14 telemetered free-ranging greater sea snakes, *Hydrophis major* (colored lines), as a function of tidal flow (hourly rate of change in height of tide) during day and night and across seasons in shallow inshore bays near Noumea, New Caledonia. Tidal height categories <0 indicate the falling phase of the tide and values >0 indicate the rising phase. Means and standard errors of numbers of movements across all individuals are shown (black points) for each category across seasons and times of day.

**Fig 5:**
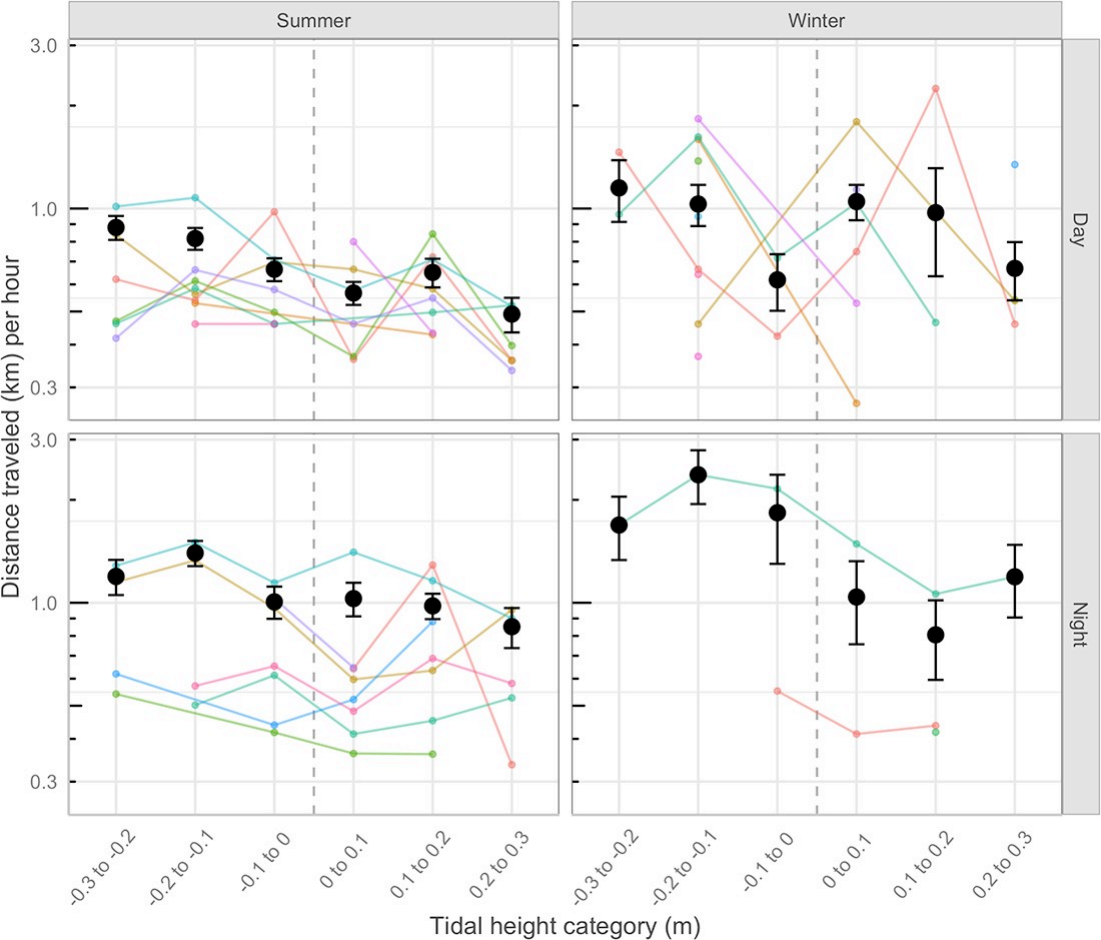
Distance traveled by snakes. Hourly distances traveled by 14 telemetered free-ranging greater sea snakes, *Hydrophis major* (colored lines), as a function of tidal flow (hourly rate of change in height of tide) during day and night and across seasons in shallow inshore bays near Noumea, New Caledonia. Tidal height categories <0 indicate the falling phase of the tide and vice versa. Means and standard errors of numbers of movements across all individuals are shown (black points) for each category across seasons and times of day.

Sample sizes (numbers of detections) from 14 of the 19 tracked sea snakes were sufficient to conduct statistical analyses. In some cases, sample sizes were small, resulting in considerable variation in relationships between movement patterns and tidal conditions (Figs 4 and 5). Nonetheless, broad patterns were apparent. Although movements were recorded across the full range of tidal cycle, the snakes moved most often, and farthest, when the tide was running out rapidly, and they reduced their activity as the tide began to flow in. The number of movements by individuals within each hour of the tracking period was significantly higher during rapidly falling tides (-0.3 to -0.2 tidal height category; Fig 4, Table 1) than at other times, across seasons and times of day. Similarly, snakes moved farther during falling tides (Fig 5, Table 1). Movement rates and distances were marginally greater by night than by day, and were higher during winter than summer (Figs 4 and 5, Table 1).

**Table 1:** Summary of coefficient estimates for snake movements and distances traveled.

Parameter	Number of movements per hour (estimate ± SE)	Distances moved per hour (m, estimate ± SE)
(Intercept)	0.32 ± 0.13	-0.48 ± 0.12
Tidal category: -0.2 to -0.1	-0.02 ± 0.10	0.02 ± 0.10
Tidal category: -0.1 to 0	**-0.26 ± 0.11**	-0.21 ± 0.11
Tidal category: 0 to 0.1	**-0.29 ± 0.10**	**-0.30 ± 0.10**
Tidal category: 0.1 to 0.2	**-0.28 ± 0.10**	**-0.27 ± 0.10**
Tidal category: 0.2 to 0.3	**-0.45 ± 0.11**	**-0.47 ± 0.11**
Season: Winter	**0.67 ± 0.09**	**0.54 ± 0.09**
Time of day: Night	**0.49 ± 0.06**	**0.39 ± 0.06**
N	114	114
N (snakes)	14	14
R^2^ (fixed)	0.18	0.14
R^2^ (total)	0.27	0.21

Coefficient estimates for fixed effects in linear mixed effects models comparing numbers of movements and distances moved by 14 telemetered sea snakes across tidal height categories, seasons, and time of day. Parameters include intercept, correction factors and slopes for the random intercept linear mixed model with explanatory variable ln-transformed. Boldface font estimates indicate parameters significantly (*p* < 0.05) different to those recorded for individuals monitored during strongly falling tides in summer during the day; the numbers (and standard errors) in the Table show deviations from the mean values for snakes monitored under those reference conditions (i.e., strongly falling tides by day).

## 4. Discussion

Our studies on captive snakes suggest that greater sea snakes can detect waterborne chemical cues from striped eel catfish, and that the snakes respond to that chemical stimulus by increasing activity levels. In the wild, these snakes were most active during rapidly falling tides. This is the phase when chemical cues from hidden catfish swarms are most likely to be available. We found no evidence that catfish avoid (or select) shelter-sites scented with snake cues.

The logistical problems associated with studying large, uncommon, and venomous sea snakes mean that experimental behavioral-ecology research on these animals is virtually non-existent [38]. Although the increasing availability of acoustic transmitters can clarify behavior in free-ranging animals [31, 36, 39, 40], there are substantial logistical challenges to conducting experimental (manipulative) studies on captive snakes, let alone free-ranging animals. Nonetheless, our preliminary trials revealed some strong patterns that provide a basis for future more ambitious research.

The increased activity of sea snakes exposed to waterborne chemical cues of their prey likely reflects a foraging response. Extensive studies, primarily on natricine colubrids, have shown that aquatic snakes respond to cues of prey items (fish, tadpoles) with increased rates of tongue-flicking and investigatory behavior [41]. The same is true for many terrestrial snakes [42]. Studies on non-natricine aquatic snakes have reported that chemical cues from the prey initiate foraging responses in some taxa (e.g., acrochordids [37]) but not others (e.g., the homalopsine *Pseudoferania polylepis* [7]). Data on sea snakes are limited, but some taxa have been reported to use chemical cues from their prey to select foraging sites (*Emydocephalus annulatus* [20]; *Hydrophis melanocephalus* and *H*. *ornatus* [21]). For logistical reasons, we tested only catfish-scented versus control water, rather than including multiple other treatments (such as pungency controls and natural chemicals with no biological significance to the test subjects). Responses to such latter cues are difficult to interpret without calibration for concentration effects [43]. Given that snakes of many phylogenetic lineages are able to detect chemical cues from prey, and that catfish are covered by thick mucus (and hence, are likely to emit more chemical cues than do some other kinds of prey), catfish likely are readily detectable by predatory snakes.

For any negative result, such as the lack of avoidance of snake cues by catfish in our study, we need to ask if the result is biologically valid, or alternatively is due to methodological problems. The skin-based chemicals that snakes use to recognize conspecifics tend to be large water-insoluble lipids [44], and relatively few of these lipids would be rubbed off on moistened paper (many researchers use a solvent like hexane to obtain such samples [16]). The morphology of the snake epidermis means that it will release far fewer chemical cues than would the mucus that coats a catfish [45]. Nonetheless, our vigorous rubbing should have transferred at least some chemical cues [30], and our experimental design (placing cheesecloth within a small shelter) should have generated high concentrations of snake-derived chemicals within each shelter (especially, relative to concentrations likely to be experienced by a fish sheltering in a crevice some distance from a foraging snake in the wild). Thus, we doubt (but cannot prove) that lack of cues, or reliance on the same set of catfish for all of these trials, were significant problems. Instead, the catfish appeared not to detect, or detect but not respond to, chemical cues from predatory snakes; a strong contrast to the behaviors that have been recorded in many terrestrial prey types (e.g., lizards [46, 47]). In those cases, however, chemical cues deposited on the substrate provide long-lasting, concentrated stimuli that provide strong evidence of a snake’s proximity. Avoidance responses by prey tend to be stronger to chemical cues from ambush-foraging snakes [12] rather than active-foraging snakes (such as *H*. *major*), because cues deposited by a wide-ranging snake may not predict its current location [37]. Also, the optimal response by catfish to cues from snakes may be crypsis; because fleeing may alert the snake to the presence of prey, and escape may be difficult (especially as water levels fall during the ebb tide). Once a snake has determined the approximate location of a catfish, other sensory modalities may come into play both for the predator (e.g., mechanoreceptors that detect water vibrations [48, 49]) and the prey (e.g., electroception [9]).

Lastly, we turn to the result from our field studies: greater sea snakes were more active when the tide was running out strongly, than at other phases of the tidal cycle (Fig 4). These patterns were seen both by day and by night, and during both winter and summer. A falling tide may enhance foraging success by concentrating prey in a smaller volume of water [50]. Synchrony with tidal cycles also might enhance foraging success if the prey species shows a tidal pattern in activity and hence, availability. We have no data on tidal rhythms of activity in catfish with which to test that idea, and studies to gather such data would be useful. Although the timing of activity by snakes accords with our hypothesis based on the enhanced availability of chemical cues from potential prey items, other factors also might contribute to the link between snake activity and tidal cycle. For example, studies in Western Australia reported that *Hydrophis elegans* avoids shark predation by shifting from open habitats into seagrass beds as the tide rises [51], whereas *H*. *major* avoids the edges of seagrass beds when sharks are abundant [52]. Avoidance of predatory sharks is unlikely to be important in our study system because we have never observed large sharks at our study sites (at any tide) although large sharks certainly occur at deeper sites within both Baie des Citrons and Anse Vata. Further research could usefully explore the influence of large sharks on movement patterns of sea snakes within this area.

The cues that induce higher activity in *H*. *major* during rapidly falling tides remain unclear. This shift may be a response to an increased concentration of catfish-derived chemicals, as cues pour out from catfish shelter-sites into a decreasing volume of water, mimicking the results of our behavioral experiments. Alternatively, the snakes may respond to tidal conditions per se, based on an underlying correlation between foraging success and tidal phase. The higher numbers of snakes and greater activity of telemetered individuals observed during winter in our study closely matches seasonal variation in rates of sightings of *H*. *major* within the bay [29]. Seasonal movements are likely linked with breeding and courting behaviors (and possibly, abundance of predators and prey), but cannot explain the consistent (year-round) increased movement rates associated with falling tides. Without direct observations, it is difficult to differentiate the signatures of foraging and courting behaviors during winter. Future experimental work could tease apart those alternatives, by seeing how activity levels of snakes shift with simulated tidal phase in the absence of chemical cues from prey.

A recent resurgence of research on sea snakes is providing a far richer understanding of the biology of these animals [40], confirming that sea snakes possess a wide range of morphological and physiological adaptations to oceanic habitats [38, 53]. Increasingly, the behavioral adaptations of these animals to marine life are being revealed also [31, 39]. To integrate these insights from free-ranging animals with experimental tests of causal mechanisms, investigators will need to find ways to manipulate cues available to snakes in natural or semi-natural conditions. Our preliminary work (and see [17]) suggests that sea snakes may be amenable subjects for experimental approaches, reacting in interpretable ways to stimuli (such as chemical cues) manipulated by the experimenter. Studies of snake responses to other stimuli, such as visual cues of potential food items, mates or predators should be equally feasible. The logistical obstacles are considerable, but we need experiments of this type if we are to shift from simple lists of prey items for each species of sea snake, to a more nuanced understanding of how these enigmatic predators and their prey interact in the marine environment.

## Supporting information

S1 Video Sea snake foraging. Footage of a greater sea snake (*Hydrophis major*) capturing a striped eel catfish (*Plotosus lineatus*) at Baie des Citrons, New Caledonia. Video by Aline Guémas.
(MP4)
